# Using strengths to attack weaknesses – The effect of comparative advertising on purchasing intention of green products

**DOI:** 10.3389/fpsyg.2022.1051856

**Published:** 2022-11-10

**Authors:** Kuocheng Ni, Yanfeng Lin, Shenghong Ye, Zhiheng Lin, Yunxiao Liu

**Affiliations:** ^1^Education Calligraphy and Painting Association, Beijing, China; ^2^School of Management, Jinan University, Guangzhou, China

**Keywords:** green product, comparative advertising, advertising appeals, green involvement, perceived diagnosticity of information

## Abstract

Firms increasingly use comparative advertising in green marketing to convey information of green products to consumers, but there is still a lack of research on the effect and mechanism of comparative advertising in the green products field. Across four experimental studies, we show that comparative advertising facilitates consumers’ purchase intention of green products (PIGP), because comparative advertising lead to higher perceived diagnosticity of Information. Yet, comparative advertising does not always bring high intention to buy green products. When using egoistic appeals, the perceived diagnosticity of information and purchase intention of green products were higher in comparative advertising than in non-comparative advertising. When utilizing altruistic appeals, there was no significant difference between the two kinds of advertising. In addition, individual differences of consumers also affect the effect of comparative advertising. The positive effect of comparative advertising on the purchase intention of green products is weakened for consumers with high green involvement. Our findings advance existing knowledge about the use of comparative advertising in green marketing and provide enlightening suggestions for how firms can promote consumers to buy green products.

## Introduction

In recent years, the environmental pollution problems caused by human production and living activities are very prominent. It is necessary to better understand and identify the methods that affect human behavior to improve these problems (Schneider et al., 2001). One of the means is to encourage consumers to engage in green consumption and choose or buy green products instead of conventional products (White et al., 2019; Tezer and Bodur, 2020). How to take effective measures to promote consumers from traditional consumption behavior to green consumption behavior has become an important social issue. More and more firms change their strategies, actively manufacture and promote green products that can meet the green needs of consumers and improve the ecological and environmental benefits (Leonidou et al., 2011).

Compared with ordinary products, green products not only have the functional attributes for normal use by consumers, but also have more prominent environmental attributes. The raw materials or production processes of green products bring less pollution to the society and ecological environment (Mazar and Zhong, 2010). In business practice, many advertisements of green products mention products of “other brand” or similar conventional products, comparing the functional attributes or environmental attributes of products to derogate the disadvantages of conventional products or highlight the advantages of green products. This is the application of comparative advertising in the context of green consumption. For example, Australia’s Morning Fresh concentrated detergent compares itself with “other brands” in terms of ingredients, test standards, uses and environment-friendly attributes in its advertisements to highlight the advantages of its own products. In the advertisement, Ecover eco-friendly dishwashing liquid compares itself with conventional products, conveying the information about functional attribute and environmental attributes: “conventional detergent contains harmful petrochemical components and hurts hands, while Ecover dishwashing liquid is extracted from natural plants, safe and mild…the bottles of Ecover dishwashing liquid are made of 100% renewable materials, which reduce 70% carbon emissions compared with other conventional products using original plastic.”

However, there is no definitive conclusion on the effect of comparative advertising, which needs to be analyzed according to the specific conditions, and there is a lack of discussion on comparative advertising in the field of green consumption. To address this situation, we focus on design dimension (i.e., advertising appeals), psychological processes, and consumer-specific condition of comparative advertising used for green products. We intend to answer the following questions important for the successful design and presentation of advertisements for green products: Can comparative advertising effectively attract consumers to buy green products? If so, what are the key dimensions in comparative advertising that encourage consumers to buy green products? What are the relevant underlying psychological processes and boundary conditions?

Based on Accessibility—Diagnosticity Theory (Feldman and Lynch, 1988; Lynch et al., 1988), we hypothesize that compared with non-comparative advertising, comparative advertising will facilitate consumers’ purchase intention of green products (or PIGP). Further, we predict that such effect occurs through a process of consumers’ perceived diagnosticity of advertising information. However, we do not expect these predictions to hold when altruistic appeals are used or green involvement of consumers is high. Four experiments across various product categories provide conclusive evidence for our theory.

Our research makes several contributions to the existing literature. First, we expand the research perspective of comparative advertising and enriched the literature of comparative advertising. By reviewing and summarizing the literature on comparative advertising, we find that the existing research has not reached a consistent conclusion on how comparative advertising affects consumers, that is, the effect of comparative advertising is different in different conditions. More importantly, comparative advertising in the field of green consumption has not received enough attention from scholars. Our research discusses the positive effects of comparative advertising comparing green products with “other brands” or similar conventional products in the context of green product marketing. Besides, we also elaborate the psychological mechanism of comparative advertising to enhance consumers’ intention to buy green products, and reveal the moderating role of advertising appeals and green involvement on the effect of comparative advertising.

Second, our findings advance earlier studies that provide limited insights into the appeals of green advertisements. According to the information of green products’ functional and environment-friendly attributes conveyed in comparative advertisements, our research classifies the advertising appeals into egoism and altruism, and verifies that in the context of expressing egoistic appeals, the use of comparative advertisements has a more positive impact on consumers’ PIGP than non-comparative advertisements; when expressing altruistic appeals, there is no significant difference between the effect of comparative advertising and non-comparative advertising on consumers’ PIGP.

Third, by finding that comparative advertising based on “other brands” products or conventional products has a positive impact on the PIGP, our research provides a theoretical reference for firms to make effective green product advertising strategies. In particular, we also show that comparative advertising play a more positive role in low-green-involvement consumers who lack knowledge of green products. Therefore, in the marketing practice, according to the insight of the target consumers and product attributes, firms can use comparative advertising to better convey the green product information by comparing with the conventional products familiar to consumers or products of “other brands.”

Finally, our research provides firms with valuable insight on how to successfully design and present comparative advertisements of green products. We test the interaction between advertising types and advertising appeals, and reveal that when egoistic appeals are adopted, comparative advertising leads to more positive PIGP than non-comparative advertising; and the adoption of altruistic appeal cannot produce this difference. Therefore, even though comparative advertising can facilitate consumers’ purchase intention, it is not enough for firms to rely on altruistic information such as environmental protection, recycling and low carbon in advertising design to attract consumers. Instead, they should highlight the functional attributes of green products different from other products, so as to truly benefit from the use of comparative advertising strategies.

## Theoretical background and hypothesis development

### Green products and green consumption

Green products refer to products that have little or no negative impact on the environment at various stages such as R&D, production processes, use and post-use disposal (Organisation for Economic Co-operation and Development (OECD), 2002). There are differences between green products and conventional products in terms of functional and environment-friendly attributes. On the one hand, the raw materials, production processes and product functions of green products are high innovative (Lao, 2014). On the other hand, green products have stronger environment-friendly attributes, effectively reducing pollution and damage to the ecological environment (Gershoff and Frels, 2015).

In daily life, purchasing clean products without harmful petrochemical components, healthy organic food, energy-saving household appliances and electric vehicles are all green product purchases. Previous studies show that intention can predict behavior in various situations (Mullet and Karson, 1985). In the field of green consumption, PIGP can well predict the actual green purchase behavior of consumers (Ajzen, 1991). Consumers who show positive PIGP are more likely to take green product purchase behavior. Our research considers that PIGP is the psychological tendency of consumers to choose and buy green products actively, and is the conscious guidance of the actual purchasing behavior of green products. We use PIGP as the predictor of the purchasing behavior of green products.

### Comparative advertising

Comparative advertising is the advertisements in which advertisers compare their own brands and products with those of other competitors in an explicit or implicit way to highlight their own advantages and thus influence consumers’ purchase decisions and other consumption behaviors (Romano, 2004). “Competitor” (with whom to compare), “competitive product or service” (what to compare) and “comparison” (how to compare) are the three key elements of comparative advertising.

According to whether contain specific competitive brand or product information, comparative advertising can be classified into direct comparative advertising and indirect comparative advertising (Miniard et al., 2006; Bambauer-Sachse and Heinzle, 2018). As the name implies, the direct comparative advertising refers to the advertisement in which a brand or product compares with the named competitors (Pechmann and Ratneshwar, 1991). Indirect comparative advertising refers to the comparison with the anonymous competitors, such as products of “other brands” or “other similar products” (Miniard et al., 2006). It is worth noting that although comparative advertising has been used widely, direct comparative advertising is controversial and prohibited in some countries because it is easy to infringe the interests of the compared object (Manzur et al., 2012). On the contrary, indirect comparative advertising helps firms avoid possible legal risks and is more common in marketing practice. Therefore, our research focuses on indirect comparative advertising of green products.

Previous studies have shown that comparative advertising is better than non-comparative advertising in improving consumers’ attention, interest, information recall and brand attitude (Beard, 2016; del Barrio-García et al., 2020). One of the important driving factors may be activation (Bambauer-Sachse and Heinzle, 2018). Activation represents the level of internal energy mobilization and excitation caused by environmental stimuli such as advertisements (Yan et al., 2016). The information provided by comparative advertising is often considered unique, valuable and personal, leading to a high level of activation. A high degree of activation leads to more attention, enhances information processing (Storbeck and Clore, 2008), and affects emotion and cognition (Gorn et al., 2001). On the other hand, comparative advertising may trigger negative reactions (Beard, 2016; Bambauer-Sachse and Heinzle, 2018; del Barrio-García et al., 2020). Consumers often believe that comparative advertising is more aggressive, less credible (Barone et al., 2004) and more manipulative (Chang, 2007) than non-comparative advertising. Comparative advertising can also cause refutation and derogation (Pant et al., 2014). In summary, there is no consistent conclusion on the impact of comparative advertising on consumers. In different conditions, comparative advertising may produce different effects. More importantly, researchers still lack attention to comparative advertising in the context of green consumption. We propose that comparative advertising can promote consumers’ PIGP.

### The effect of comparative advertising on PIGP

Comparative advertising provides specific attribute information of products (such as feature, quality and advantages) so that consumers can make inferences based on similarities or differences (Dröge and Darmon, 1987; Pechmann and Ratneshwar, 1991). The comparative advertisement of green products does not make a comparison directly between a green product and another specific brand or product, but indirectly compares with a product of “other brands” or similar conventional products to publicize the comparative advantages of green products to win market share. Indirect comparison is considered to be more objective than the direct comparison.

The relationship between green products and traditional products is one of substitution and competition, which has relevance and comparability and conforms to the elements of comparative advertising. Moreover, green products show obvious advantages over conventional products: on the one hand, green products have certain innovation in raw materials, manufacturing processes, efficacy and performance (Lao, 2014), which means that the functional attributes of green products may be better than those of conventional products; on the other hand, the production process of green products has little or no negative impact on the environment (Gershoff and Frels, 2015), so it has more environment-friendly attributes than conventional products. Because the advantages of green products are obvious and widely recognized, the use of comparative advertising for green products is not easy to bring undesirable outcomes such as consumers’ refutation and resistance. Therefore, it seems appropriate and beneficial to use comparative advertising for green products.

We believe that green products can get more attention by using comparative advertising. First, more than one product is mentioned in the advertisements, which increases the personal relevance to more consumers (Wilkie and Farris, 1975) and attracts more consumers’ attention (Grewal et al., 1997). Second, comparative advertising provides more information (Muehling et al., 1990). Comparative advertising displays both the positive information of green products and the negative information of conventional products at the same time, which leads to higher stimulation (Pechmann and Stewart, 1990) and more information processing (Muehling et al., 1990). Compared with green products, consumers are generally more familiar with conventional products and have more use experience. Comparative advertising may activate the pre-existing knowledge structure of consumers and enhance the availability of advertising information, which is conducive to consumers’ positive brand attitude and high willingness to use products (del Barrio-García et al., 2020). We hypothesize:

*H1*: Comparative advertising leads to higher PIGP than non-comparative advertising.

### Accessibility-diagnosticity theory and perceived diagnosticity of information

Accessibility-diagnosticity theory (Feldman and Lynch, 1988; Lynch et al., 1988) proposes that whether information can be used by consumers for cognitive evaluation and decision depends on the accessibility and diagnosticity of information. Accessibility refers to how easy it is for consumers to recall and extract relevant information in their own memory, or how easy it is for certain information to be extracted and used relative to other information (Anderson, 1983). Diagnosticity refers to the usefulness of information in the cognitive and decision evaluation. The stronger the correlation between the information and the evaluated object, the more useful the information is for cognitive evaluation, that is, the higher the diagnosticity (Skowronski and Carlston, 1987; Dick et al., 1990).

The usefulness of information depends on the subjective perception of consumers. If consumers think that the information is helpful for them to know more about the product and make purchase decisions, they will use it to evaluate the product, so the consumers perceive the information as highly diagnosable. In particular, when little information is provided or the consumers have little relevant knowledge, the perceived diagnosticity of the existing information will be high (Feldman and Lynch, 1988; Lynch et al., 1988). Filieri’s research (Filieri, 2015) shows that improving consumers’ perceived diagnosticity of certain information can promote them to make positive purchase decisions, because higher perceived diagnosticity of information can make consumers feel that they have a high understanding of the product and have greater confidence in their purchase decisions.

For an advertisement of a green product, the consumers’ perceived diagnosticity of information refers to the extent to which consumers think that the advertising information is helpful to evaluate the green product and make decisions. First, compared with non-comparative advertising that only provide green product information, the introduction of conventional products information that consumers are relatively familiar with in comparative advertising can activate the pre-existing knowledge structure of consumers and help them extract relevant information from situations or memories, that is, the accessibility of comparative advertising information is higher. Secondly, comparative advertising compares the advantages and disadvantages of green product with conventional products or “other brand” products in terms of functional attributes or environmental attributes, providing consumers with detailed available information to make consumption decisions. Moreover, comparative advertising also provides reference points in the process of information coding (Lien, 2001), which can enhance consumers’ ability to process and understand information (Moorman, 1990), and reduce consumers’ doubts through more rational thinking. Thus, consumers lacking knowledge of green products can benefit from the benchmark provided by reference information (Lien, 2001).

Therefore, the information provided by comparative advertising is more relevant to decision-making than non-comparative advertising, which can better help consumers understand and evaluate products. That is, the consumers’ perceived diagnosticity of comparative advertising information is high. And high perceived diagnosticity can promote consumers’ positive evaluation of product information and improve consumers’ intention to buy green products. More formally:

*H2*: The influence of comparative advertising (vs. non-comparative advertising) on consumers' PIGP is mediated by perceived diagnosticity.

### Egoistic and altruistic appeals in green advertisement

For the advertisement of green products, egoistic appeals emphasize the benefits that green products can bring to consumers, and explain the products functional features to let consumers know the health benefits and economic benefits that they can obtain (Green and Peloza, 2014; Kareklas et al., 2014). On the contrary, altruistic appeals emphasize the benefits that green products can bring to the environment. By describing the environmental attributes of green products, altruistic appeals enable consumers to know the benefits that choosing green products can be generated for the society and the natural environment (Kareklas et al., 2014). The comparative advertising of green products usually points out the disadvantages of conventional products in terms of efficacy and performance to publicize the benefits brought by green products to consumers, which expresses egoistic appeals; or it claims the degree of damage caused by conventional products to the environment and emphasizes the effect of green products on environmental protection (Mazar and Zhong, 2010), which expresses altruistic appeals.

The influence of egoistic and altruistic appeals on consumers’ purchase intention can be explained by Elaboration Likelihood Model (Petty and Cacioppo, 1986). According to the Elaboration Likelihood Model, there are two different paths for people to process the received information – the central route and the peripheral route. Under the central route, people think about the information comprehensively and fully, actively match their existing knowledge and experience with the received advertising information, and then make reasoning and judgment. If the product information in the advertisement is consistent with one’s own product knowledge and past experience, he/she is likely to have a positive attitude towards the advertised product. Under the peripheral route, people do not think deeply and rationally about the information in the advertisement, but pay more attention to peripheral cues in the advertisement and make decisions according to their emotional reactions. Whether to choose the central route or the peripheral route mainly depends on the motivation and ability of individuals to think about the information. When individual has strong motivation and enough analytical ability, he/she will process information and make decisions *via* the central route; otherwise, one will process information and make decisions *via* peripheral route (Allison et al., 2017).

In green advertising, egoistic appeals are generally based on the functions of green products, highlighting the benefits of using green products on consumers’ personal health, use experience, efficiency improvement, etc. These appeal points are more relevant to individuals and the accessibility of information is higher. Thus, before purchase decision-making, consumers are more inclined to carefully consider and logically judge advertising information *via* the central route (Lee et al., 2017). Under these conditions, compared with non-comparative advertising, comparative advertising provides detailed information of green product and conventional product at the same time, making consumers realize that the advertising information conforms to their own interests and helps them make purchase decisions. Therefore, we predict that for egoistic appeals, comparative advertising increases consumers’ perceived diagnosticity of information, which leads to positive PIGP.

And altruistic appeals are based on the environmental attribute of green products, describing the impact of green products on the ecological environment and other people’s interests. These appeal points are less relevant to individuals, and the accessibility of information is low. Consumers are more inclined to deal with the product information in advertisements *via* the peripheral route. In this case, consumers pay less attention to the altruistic information in the advertisement, and the diagnosticity of the advertisement information is low (Xie et al., 2015). Therefore, for altruistic appeals, consumers are unlikely to carefully analyze the information describing the environmental attributes of products in advertisements, leading to no significant difference in their perceived diagnosticity of advertising information and PIGP between comparative advertising and non-comparative advertising. We propose:

*H3*a: When using egoistic appeals, comparative advertising leads to higher perceived diagnosticity and PIGP than non-comparative advertising.

*H3*b: When using altruistic appeals, there is no significant difference between the influence of comparative advertising and non-comparative advertising on consumers' perceived diagnosticity and PIGP.

### Moderating role of green involvement

Green involvement is an individual’s own interest and engagement in green brands, green products and related green information. Individuals with higher interests in green products are more willing to read green product advertisements and reports on natural environment issues. They have a better understanding of green product knowledge and the importance of environmental protection, who are called “high-green-involvement consumers,” and conversely, “low–green-involvement consumers.” Grimmer and Bingham (2013) found that consumers with high green involvement have more positive attitudes towards green products and related information, while the attitudes of consumers with low green involvement are negative or indifferent. However, De Vlieger et al. (2012) proposed that there is a negative correlation between consumers’ green involvement and green product trust. The lower the green involvement, the more likely consumers are to show a higher degree of trust in green product information. Although scholars do not reach a consensus on the pattern of green involvement influencing consumers, green involvement is an important factor affecting consumers’ evaluation of green products.

Consumers high in green involvement are interested in green products and relevant environmental protection information, have rich green product experience and knowledge, and are more likely to be convinced by green advertising information (Haytko and Matulich, 2008). Therefore, no matter whether comparative advertising or non-comparative advertising is used for green products, consumers with high green involvement will rationally call their own knowledge structure, actively process the advertising information of green products *via* the central route (Xu et al., 2015). And they compare and evaluate green products and conventional products from the perspective of functions and value to help them make purchase decisions.

For consumers low in green involvement, on the one hand, their interest and understanding of green products information are relatively low, and they seldom actively and extensively collect information related to green products (Haytko and Matulich, 2008). They lack experience of using green products, so they are more vulnerable to external cues (such as advertising information) when making consumption decisions. Compared with non-comparative advertising, comparative advertising containing conventional products familiar to consumers can attract consumers’ attention and guide them to process more advertising information. Moreover, directly presenting the comparative information between green products and conventional products in advertisements reduces the extra information search efforts of consumers, which is more helpful for consumers with low green involvement to make consumption decisions. On the other hand, consumers with low green involvement usually use the peripheral route to process green information without strict logical reasoning and information comparison. They are easy to make more emotional responses according to advertisements and pay more attention to the resonance effect between product information and their own emotions, resulting in their positive purchase intention for green products.

*H4*a: When consumers' green involvement is high, there is no significant difference in the effect of comparative advertising and non-comparative advertising on consumers’ PIGP.

*H4*b: When consumers' green involvement is low, comparative advertising leads to more positive PIGP than non-comparative advertising.

**Figure 1:**
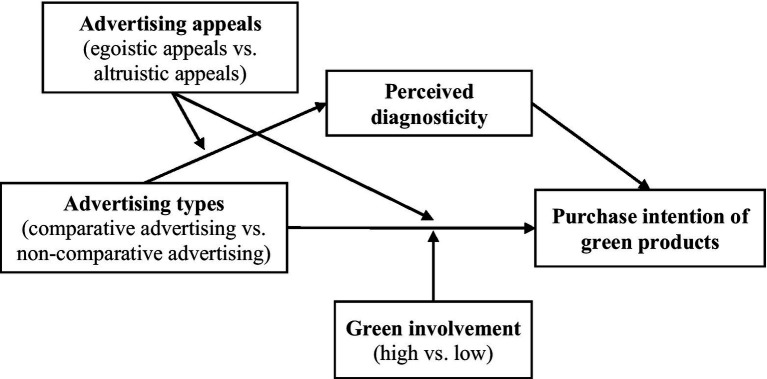
Conceptual model.

In the following, we test our hypotheses through four studies. Study 1 preliminarily examines the main effect of comparative advertising (*H1*) based on the comparison with products of “other brands.” Using a new category of green products, Study 2 tests the main effect of comparative advertising (*H1*) and the mediating role of perceived diagnosticity (*H2*) in the context of comparison with conventional products. Study 3 examines the moderating effect of advertising appeals by manipulating the egoistic and altruistic appeals expressed in advertisements. Study 4 tests the moderating effect of green involvement on the positive impact of comparative advertising by measuring consumers’ green involvement (Figure 1).

## Study 1: The effect of comparative advertising on PIGP

The purpose of Study 1 is to test the effect of comparative advertising on consumers’ PIGP in the context of comparing green products with products of “other brands.”

### Design and participants

Study 1 utilized a two-level (advertising types: comparative advertising vs. non-comparative advertising), one-way between-subjects experimental design. Sixty-four participants were recruited from Credamo^1^ in exchange for monetary compensation and randomly assigned to one of two conditions. Of these participants, 4 were removed from final analyses because they failed to complete the study in its entirety. Thus, the final sample consisted of 60 participants (*M*_Age_ = 25.78, *SD*_Age_ = 4.62; 58.33% female).

### Procedure and stimuli

The choice of experimental stimuli and the manipulation of comparative advertising refer to previous research on green products and comparative advertising (Choi and Miracle, 2004; Miniard et al., 2006; Chang et al., 2015). In Study 1, the green product, environment-friendly dishwashing liquid, was used as the experimental stimulus, and the real brand Morning Fresh was selected to manipulate the advertising types in the form of pictures. First, the participants were asked to imagine that they needed to buy detergent in the near future, and then read the advertisement of Morning Fresh environment-friendly dishwashing liquid carefully.

In the comparison advertising condition, the participants read “Brand Comparison – You are right to choose Morning Fresh!” and comparative information of the products of Morning Fresh and “other brands” in the picture, including the use, composition, environmental attributes and so on. In the non-comparison advertising condition, the participants read “Brand Introduction – You are right to choose morning fresh!” and information about Morning Fresh such as the use, composition, environmental attributes. In addition to product information, there is no difference in other features of pictures in both conditions.^2^

After reading the advertisement, the participants answered the attention check: “What product(s) is (are) mentioned in the advertising content you just read? 1 = Morning Fresh dishwashing liquid, 2 = Morning Fresh dishwashing liquid and dishwashing liquids of other brands.” They also reported their purchase intention of green products (Lee et al., 2008) with three items: “I want to buy the product,” “it is a wise choice to buy the product in the advertisement,” “the green advertisement prompts me to buy the product” (1 = strongly disagree, 7 = strongly agree; *α* = 0.82). Considering the possible impact of the real brand, we assessed participants’ brand familiarity (*α* = 0.91) and brand evaluation (*α* = 0.75). Brand familiarity was assessed with four items (Campbell and Keller, 2003): “I often see advertisements about this brand,” “I often see the display or sale of this brand’s products,” “I often hear others talk about or recommend this brand,” “I often buy or use this brand’s products” (1 = strongly disagree, 7 = strongly agree). The brand evaluation scale (Thompson and Malaviya, 2013) haves three items: “your overall impression of the brand is: (1 = “very bad / very low quality / quite dislike “, 7 = “very good/very high quality/quite like”). Finally, participants answered demographic variables such as gender and age.

### Results

#### PIGP

**Figure 2:**
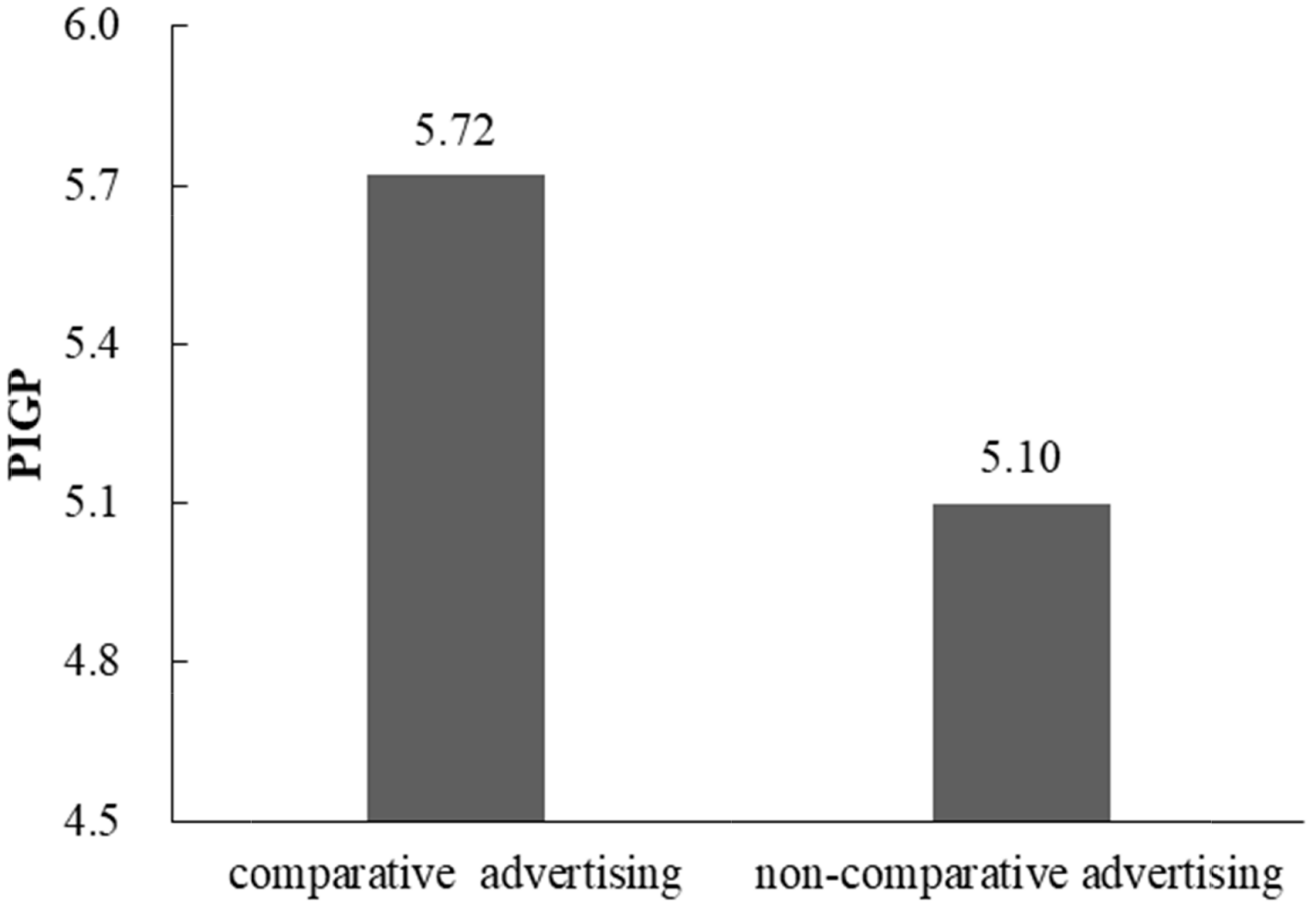
PIGP for comparative advertising and non-comparative advertising.

Comparative advertising condition was coded as 1, and non-comparative advertising condition was coded as 2. A t-test showed that participants in comparative advertising condition reported higher PIGP (*M* = 5.72, *SD* = 0.70) than in non-comparative advertising condition (*M* = 5.10, *SD* = 0.94; t (58) =2.89, *p* < 0.01, Cohen’s d = 0.93; Figure 2).

In addition, gender had a significant main effect on PIGP (*F* (1, 58) = 4.52, *p* < 0.05). An ANOVA with gender as the covariant revealed that, the effect of comparative advertising on PIGP was still significant (*F* (1, 57) = 8.34, *p* < 0.01, partial *η*^2^ = 0.13). Apart from the reported results, no other significant main effects were found.

### Discussion

Study 1 preliminarily supports hypothesis 1, that is, comparative advertising can lead to higher intention to buy green products than non-comparative advertising. However, Study 1 still has shortcomings. For example, brand familiarity of consumers may confound our results because of the real brand used in the experiment. To this end, we designed Study 2, which uses energy-saving air conditioner as a new stimulus, and create a fictional brand to avoid unnecessary interference. In the context of comparing with conventional products, we examine the impact of comparative advertising on consumers’ purchase intention of green products again, and explore the mediating role of perceived diagnosticity.

## Study 2: The mediating role of perceived diagnosticity

Using fictional brand and new green products as stimuli, Study 2 tests the impact of comparative advertising again in the context of comparison with conventional products, and explores the mediating role of perceived diagnosticity.

### Design and participants

Study 2 employed a two-level (advertising types: comparative advertising vs. non-comparative advertising), one-way between-subjects experimental design. Sixty-six participants were recruited from Credamo in exchange for monetary compensation and randomly assigned to one of two conditions. Of these participants, 6 were removed from final analyses because they failed the attention checks. Thus, the final sample consisted of 60 participants (*M*_Age_ = 28.92, *SD*_Age_ = 5.43; 61.67% female). After analyzing green advertising in various media channels, we found that green advertising practices about household appliances and automobiles are rich. Therefore, Study 2 used energy-saving air conditioner as experimental stimulus, and created a fictional brand Nonkle to prevent the existing brand cognition of participants from interfering with the experimental results.

### Procedure and stimuli

First, participants imagined that they need to buy an air conditioner in the near future, and then carefully read the advertisement of an energy-saving air conditioner named Nonkle. The manipulation of advertising types was mainly in the form of text description. All participants read the following information: “Nonkle energy-saving air conditioner meets level-1 energy efficiency, consumes less than 1 kW•h of electricity per hour, and reduces carbon emissions,” “Nonkle energy-saving air conditioner uses high-temperature sterilization…bringing you a comfortable experience,” “Nonkle energy-saving air conditioner uses fluorine-free refrigerant to reduce the damage to the atmosphere and become the environmental protection guardian of the earth.” In the comparative advertising condition, the participants read: “Nonkle is more energy-saving than conventional air conditioners,” “Nonkle is cleaner than conventional air conditioners,” “The Freon emission of conventional air conditioners exceeds the standard and destroys the ozone layer” (Table 1). The comparative and non-comparative versions were similar except for the manipulated text information.

**Table 1:** The advertisements in different conditions.

Comparative advertising	Non-comparative advertising
Nonkle energy-saving air conditioner meets level-1 energy efficiency, which is more energy-saving than conventional air conditioners. It consumes less than 1 kW•h of electricity per hour, reduces carbon emissions, and saves money after long-term use. Nonkle energy-saving air conditioner uses high-temperature sterilization, which is cleaner than conventional air conditioners, bringing you a comfortable experience. The Freon emission of conventional air conditioners exceeds the standard and destroys the ozone layer. Nonkle energy-saving air conditioner uses fluorine-free refrigerants to reduce the damage to the atmosphere and become the environmental protective guardian of the earth.	Nonkle energy-saving air conditioner meets level-1 energy efficiency, consumes less than 1 kW•h of electricity per hour, and reduces carbon emissions, and saves money after long-term use. Nonkle energy-saving air conditioner uses high-temperature sterilization, bringing you a comfortable experience. Nonkle energy-saving air conditioner uses fluorine-free refrigerant to reduce the damage to the atmosphere and become the environmental protective guardian of the earth.

After reading the advertisement, the participants answered attention check, dependent and mediating variables. The measure of PIGP used four items on a 7-point scale (Lao, 2014): “I am willing to learn more about this green product,” “I am willing to recommend my relatives and friends to buy this green product,” “I am willing to introduce and recommend this green product to my family,” “if I need to buy it, I will buy this green product” (*α* = 0.74). In addition, perceived diagnosticity was measured with three 7-point scale items (*α* = 0.88) form Jiang and Benbasat (2007), which were adapted to fit the experiment context (“the information provided in the advertisement is very helpful for me to evaluate the product,” “the information provided in the advertisement helps me to understand the performance of the product,” “the information provided in the advertisement helps me to familiarize myself with the product”; 1 = strongly disagree, 7 = strongly agree). Finally, they reported their demographic information.

### Results

#### PIGP

**Figure 3:**
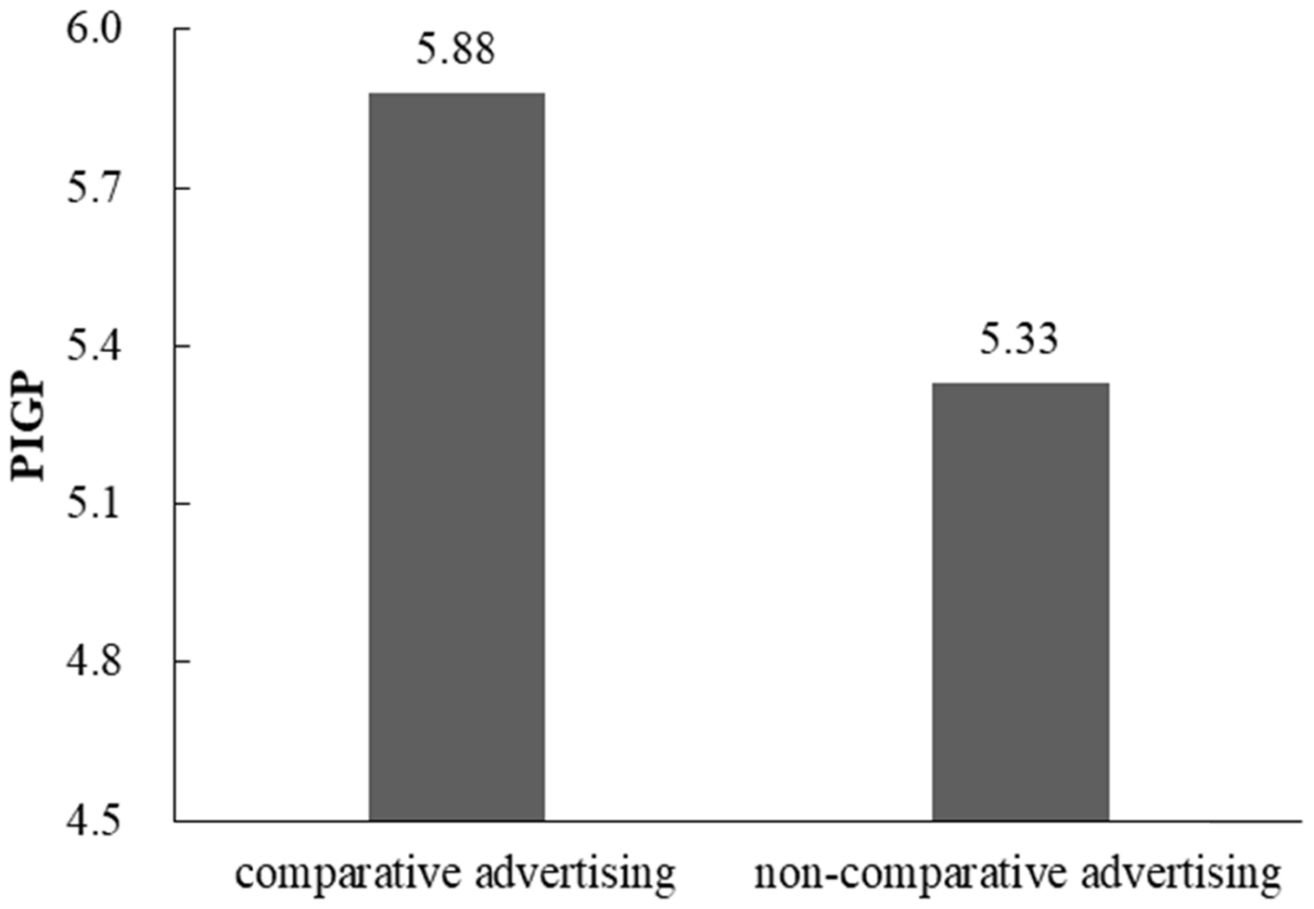
PIGP for comparative advertising and non-comparative advertising.

Comparative advertising was coded as 1, and non-comparative advertising was coded as 2. A *t*-test showed that participants reported higher PIGP in comparative advertising condition (*M* = 5.88, *SD* = 0.76) than in non-comparative advertising condition (*M* = 5.33, *SD* = 0.54; *t* (58) = 3.24, *p* < 0.01, Cohen’s *d* = 0.85; Figure 3). Apart from the reported results, no other significant main effects were found. Therefore, *H1* is still valid for green product advertisements that are compared with conventional products, that is, comparative advertising is effective to improve consumers’ willingness to buy green products.

#### Perceived diagnosticity

An ANOVA with perceived diagnosticity as the dependent variable revealed a significant main effect of advertising types (*F* (1, 58) = 9.25, *p* < 0.01, *η*^2^ = 0.14), Participants in comparative advertising condition report higher perceived diagnosticity (*M* = 5.69, *SD* = 1.04) than those in non-comparative advertising condition (*M* = 5.00, *SD* = 0.68).

#### Mediation analysis

We estimated a mediation model (SPSS Macro PROCESS, Model 4; bootstrap samples = 5,000) (Hayes, 2018) to examine whether perceived diagnosticity mediated the effect of comparative advertising on PIGP. As expected, the indirect effect was significant (*b* = −0.31, *SE* = 0.12; 95%*CI* = [−0.6180, −0.1163]), indicating that perceived diagnosticity mediated the effect of comparative advertising on PIGP, supporting *H2*.

### Discussion

With different stimuli and manipulation, Study 2 proves hypothesis 1 again in the context of comparison with conventional products. At the same time, we also verify the mediating role of perceived diagnosticity (*H2*). So far, Study 1 and 2 finds that whether green products are compared with “other brands” or similar conventional products, comparative advertising is preferable to non-comparative advertising. However, for different advertising appeals, the impact of comparative advertising on green product purchase intention will be different. In this regard, Study 3 explores the interaction between advertising appeals and advertising types.

## Study 3: The moderating role of advertising appeals

The purpose of Study 3 is to test whether egoistic and altruistic appeals can moderate the effect of comparative advertising on consumers’ purchase intention of green products.

### Design and participants

Study 3 employed a 2 (advertising types: comparative advertising vs. non-comparative advertising) × 2 (advertising appeals: egoistic appeal vs. altruistic appeal) between-subjects experimental design. We recruited 138 participants from Credamo and randomly assigned them to one of four conditions. Of these participants, 18 were removed from final analyses. Thus, analyses were run on 120 participants (*M*_Age_ = 26.93, *SD*_Age_ = 5.46; 47.50% female).

### Procedure and stimuli

Study 3 used eco-friendly detergent with a fictional brand EGW as the experimental stimulus. The non-comparative advertising condition did not mention the information of conventional detergent products. Referring to previous studies (Yang et al., 2015) on the manipulation of egoism and altruism, the comparative advertising of egoistic appeal highlighted the functional benefits of EGW detergent for consumers, involving the safety and efficiency of detergent. And the altruistic appeal of comparative advertising highlighted the environmental benefits of EGW detergent, involving the impact of detergent ingredients, packaging materials and the like on the environment. There is no obvious difference in other features between advertisements (Table 2).

**Table 2:** The advertisements in different conditions.

	Comparative advertising	Non-comparative advertising
Egoistic appeal	Chemical residues of traditional detergents are harmful to health. EGW eco-friendly detergents use natural plant ingredients, which will not irritate and hurt hands. They are little foam and easy to rinse, so as to better care for your health and that of your family. EGW eco-friendly detergent has high performance and only needs one-fifth of the amount of traditional detergent under the same effect. With various functions, EGW can save money for you for a long time.	EGW eco-friendly detergents use natural plant ingredients, which will not irritate and hurt hands. They are little foam and easy to rinse, so as to better care for your health and that of your family. EGW eco-friendly detergent has high performance and various functions, which can save money for you for a long time.
Altruistic appeal	Traditional detergents pollute the environment with a variety of chemical components. EGW eco-friendly detergents use natural plant components. The waste water after washing with EGW can be used to irrigate flowers and plants directly, which does not harm aquatic animals and plants, and can better maintain environmental safety. The bottle of EGW eco-friendly detergent is made of 100% recycled plastic, which reduces carbon emissions by 70% compared with other traditional detergents using original plastic bottles. In the long run, it can protect the ecological environment and promote sustainable development.	EGW eco-friendly detergents use natural plant components. The waste water after washing with EGW can be used to irrigate flowers and plants directly, which does not harm aquatic animals and plants, and can better maintain environmental safety. The bottle of EGW eco-friendly detergent is made of 100% recycled plastic, which reduces carbon emissions. In the long run, it can protect the ecological environment and promote sustainable development.

The participants were asked to imagine that they needed to buy detergent in the near future. After reading an advertisement for EGW eco-friendly detergent, they completed the manipulation check and attention check. We used the scale from Kareklas et al. (2014) to test if the manipulation of advertising appeals was successful. Items of altruistic appeal mainly include “the advertising content is based on environmental protection considerations/resource conservation considerations/overall social interests considerations” (1 = strongly disagree, 7 = strongly agree; *α* = 0.95). The egoistic appeal items mainly include “the advertising content is based on personal health considerations/personal use considerations/personal interests” (1 = strongly disagree, 7 = strongly agree; *α* = 0.93). Then, participants answered questions about perceived diagnosticity (*α* = 0.89; Jiang and Benbasat, 2007) and purchase intention of green products (*α* = 0.88; Lee et al., 2008). Finally, demographic information was collected.

### Results

#### Manipulation check

An ANOVA for the manipulation check of advertising appeals showed that the egoism score of egoistic appeal condition (*M* = 5.29, *SD* = 0.86) was significantly higher than that of altruistic appeal condition (*M* = 2.74, *SD* = 0.63; *F* (1, 116) = 344.63, *p* < 0.001). As for the score of altruism, the altruistic appeal condition (M = 5.44, SD = 0.59) was significantly higher than the egoistic appeal condition (*M* = 2.82, *SD* = 0.71; *F* (1, 116) = 490.76, *p* < 0.001).

#### PIGP

**Figure 4:**
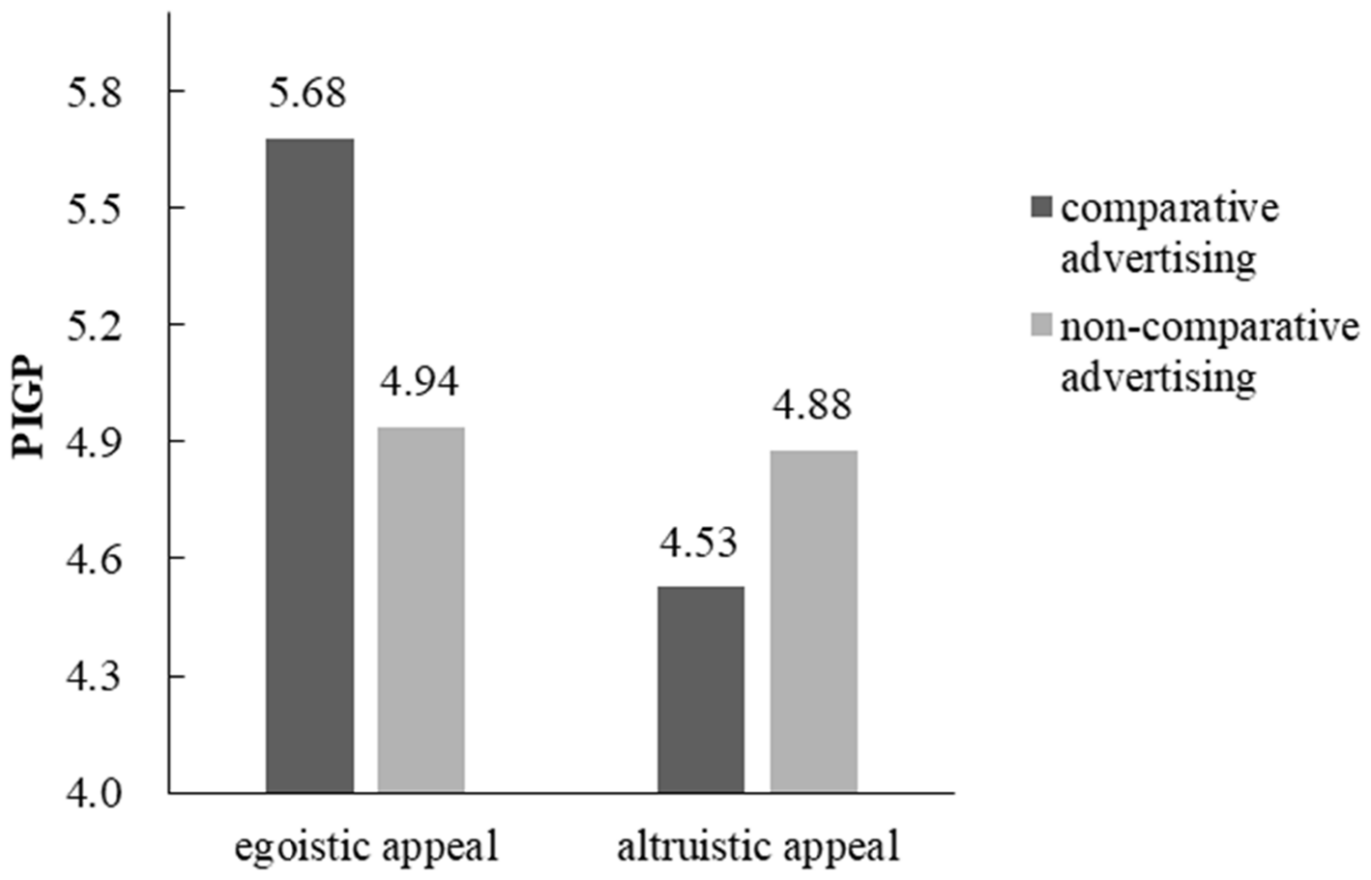
The interaction effect between advertising types and advertising appeals on PIGP.

A 2 × 2 ANOVA revealed no main effect of advertising types (coded as 1 for comparative advertising, and 2 for non-comparative advertising; *F* (1, 116) = 2.36, *p* = 0.13), significant main effect of advertising appeals (*F* (1, 116) = 23.15, *p* < 0.001, partial *η*^2^ = 0.17), and significant interaction between advertising types and advertising appeals (*F* (1,116) = 18.32, *p* < 0.001, partial *η*^2^ = 0.14). In the egoistic appeal condition, participants read comparative advertisement had higher PIGP (*M* = 5.68, *SD* = 0.64) than those read non-comparative advertisement (*M* = 4.94, *SD* = 0.52; *F* (1, 116) = 16.92, *p* < 0.001, partial *η*^2^ = 0.13). In the altruistic appeal condition, no difference in PIGP as a function of advertising types (*M*_comparative advertising_ = 4.53, SD = 0.86, *M*_non-comparative advertising_ = 4.88, *SD* = 0.69; *F* (1, 116) = 3.76, *p* = 0.055, Figure 4).

#### Perceived diagnosticity

**Figure 5:**
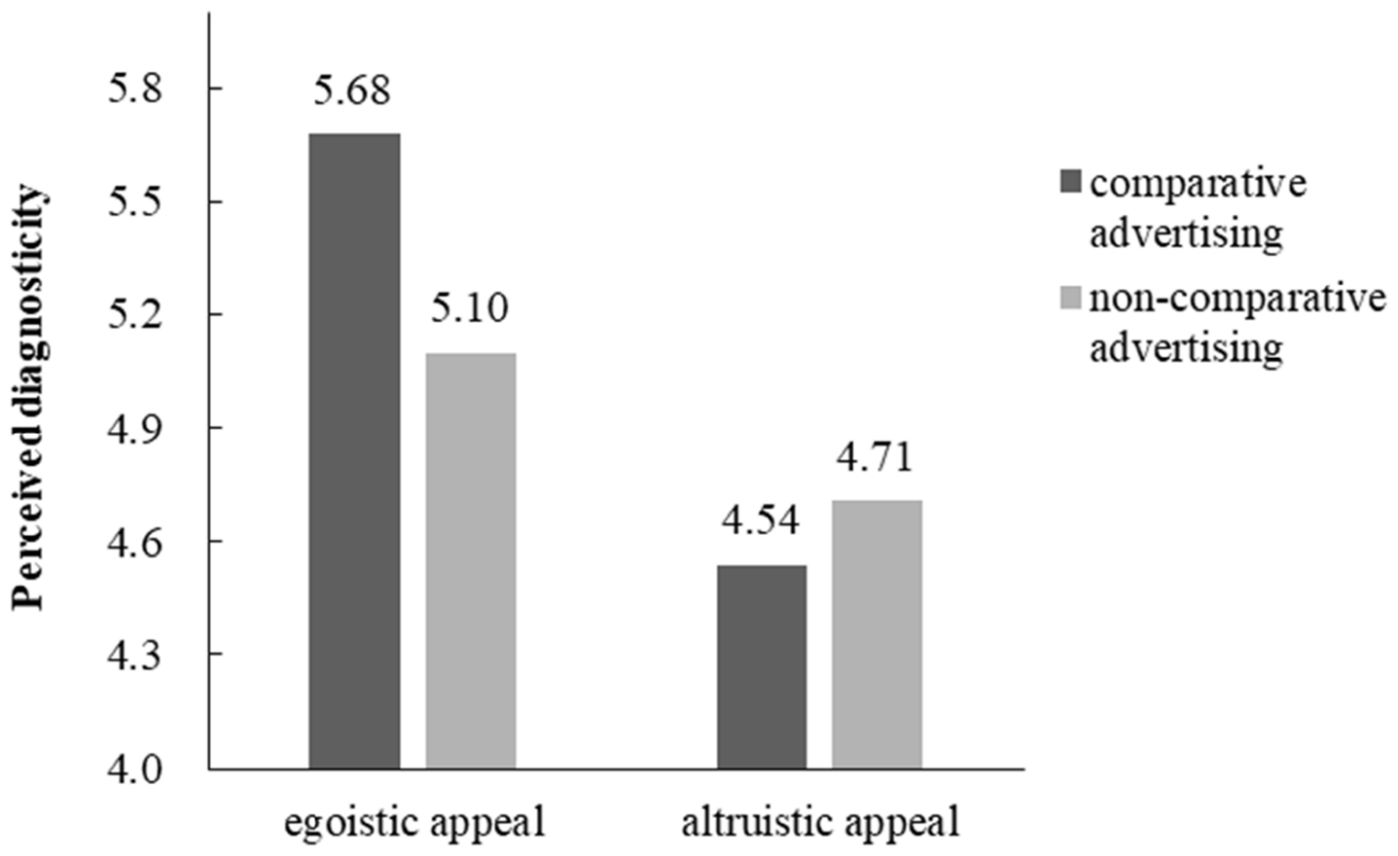
The interaction effect between advertising types and advertising appeals on perceived diagnosticity.

A 2 × 2 ANOVA revealed no main effect of advertising types (coded as above; *F* (1, 116) = 2.88, *p* = 0.09), significant main effect of advertising appeals (*F* (1, 116) = 41.13, *p* < 0.001, partial *η*^2^ = 0.26). More importantly, the interaction between advertising types and advertising appeals was significant (*F* (1, 116) = 9.69, *p* < 0.01, partial *η*^2^ = 0.08). For egoistic appeal, compared with non-comparative advertising (*M* = 5.10, *SD* = 0.53), people in comparative advertising condition (*M* = 5.67, *SD* = 0.63) reported higher perceived diagnosticity (F (*1*, 116) = 11.57, *p* < 0.01, partial *η*^2^ = 0.09). For altruistic appeal, there was no significant difference between the comparative advertising condition (*M* = 4.54, *SD* = 0.80) and non-comparative advertising condition (*M* = 4.71, *SD* = 0.59) in the perceived diagnosticity (*F* (1, 116) = 1.00, *p* = 0.32), as shown in Figure 5.

#### Moderated mediation analysis

To examine whether advertising appeals moderates the underlying process *via* perceived diagnosticity, we estimated a moderated mediation model (SPSS Macro PROCESS, Model 8; bootstrap samples = 5,000; Hayes, 2018) using advertising types (coded as above) as the independent variable, perceived diagnosticity as mediator, advertising appeals as the moderator, and PIGP as the dependent variable. The interaction between advertising types and advertising appeals was significant (*b* = 0.73, *t* (116) = 3.11, *p* < 0.01). Perceived diagnosticity, in turn, facilitated PIGP (*b* = 0.28, *t* (116) = 2.90, *p* < 0.01). Advertising appeals moderated the indirect effect of advertising types on PIGP *via* perceived diagnosticity (index = 0.20, 95%*CI* = [0.03, 0.47]). The indirect effect of advertising types on PIGP *via* perceived diagnosticity was only significant for egoistic appeal (*b* = −0.16, 95%*CI* = [−0.35, −0.03]) but not for altruistic appeal condition (*b* = 0.05, 95%*CI* = [−0.05, 0.19]), confirming *H3*a and *H3*b.

### Discussion

Study 3 reveals the interaction between advertising appeals and comparative advertising using new green products. Specifically, for egoistic appeal, comparative advertising makes consumers feel more diagnosticity of advertising information and generate more positive willingness to buy green products than non-comparative advertising. For altruistic appeal, there is no significant difference in the impact of comparative advertising and non-comparative advertising on consumers’ perceived diagnosticity and purchase intention. So far, we have explored the impact of relevant factors in advertising on consumers’ purchase intention, but the individual differences of consumers are also of great significance. Study 4 verifies how individual differences affect the effect of green product comparative advertising.

## Study 4: The moderating role of green involvement

Study 4 tests the moderating effect of consumers’ green involvement on the effect of comparative advertising (*H4*). We used environmentally friendly tissue as an experimental stimulus and created a fictitious brand BAMBO.

### Design and participants

Study 4 was a mixed-factorial design that included advertising types (comparative advertising vs. non-comparative advertising) as a between-participants factor and green involvement as the measured variable. One hundred and forty-five participants were recruited and randomly assigned to one of two conditions. The final analyses consisted 123 participants (*M*_Age_ = 27.94, *SD*_Age_ = 4.61; 55.28% female) after 22 were removed.

### Procedure and stimuli

First, we measured the green involvement (*α* = 0.93) of the participants with the scale from Wang et al. (2017) and adapted it to fix our experiment. A total of five items are included: “I am interested in reading about environmental information and descriptions of green products,” “I am willing to read consumer reports and articles about green products,” “I often pay attention to environmental information and reports or advertisements related to green products,” “I often compare the features of different green products and their impact on the environment,” “I often talk about environmental issues or green products with others” (1 = strongly disagree, 7 = strongly agree).

After answering the above questions, participants were asked to imagine that they needed to buy some tissues in the near future, and then read the advertisement (see Table 3) of bamboo pulp tissue named BAMBO (a fictional brand). In addition to product information, there is no obvious difference in other features of advertisements in different conditions. After reading the advertisement, participants filled out questions assessing purchase intention (*α* = 0.92) and demographic variables.

**Table 3:** The advertisements in different conditions.

Comparative advertising	Non-comparative advertising
BAMBO bamboo pulp tissue is made of original bamboo pulp. Compared with traditional wood pulp paper with harmful chemical ingredients, it is natural and environment-friendly, mild and no skin irritation, so that you and your family can use it safely and healthily. Traditional wood pulp tissue fells trees as raw materials. BAMBO bamboo pulp tissue uses bamboos with a short growth cycle (3–5 years) as raw materials, which is conducive to protecting forest resources, maintaining ecological environment and achieving sustainable development.	BAMBO bamboo pulp tissue is made of original bamboo pulp. It is natural and environment-friendly, mild and no skin irritation, so that you and your family can use it safely and healthily. BAMBO bamboo pulp tissue uses bamboos with a short growth cycle (3–5 years) as raw materials, which is conducive to protecting forest resources, maintaining ecological environment and achieving sustainable development.

### Results

#### PIGP

**Figure 6:**
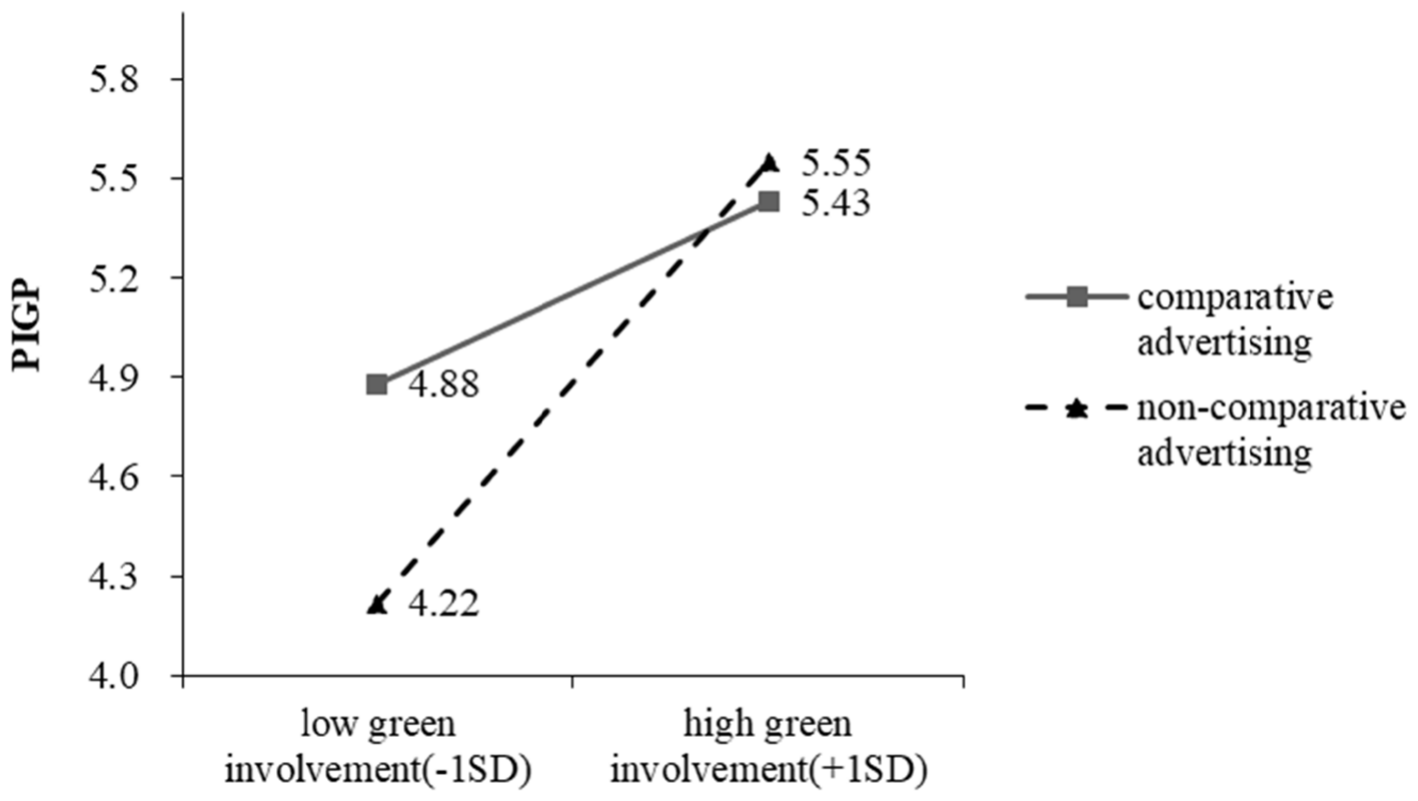
PIGP as a function of advertising types and green involvement.

To test hypothesis 4, we estimated a simple moderation model (SPSS Macro PROCESS, Model 1; bootstrap samples = 5,000)(Hayes, 2018), in which the PIGP was expressed as a function of advertising types (coded as 1 for comparative, and 2 for non-comparative), green involvement (as a continuous variable), and their interaction. The analysis revealed a main effect of advertising types on the PIGP that was negative and significant (*b* = −2.22, *t* (119) = −3.33, *p* < 0.01), which indicates that comparative advertising can promote PIGP. The main effect of green involvement on PIGP was not significant (*b* = −0.13, *t* (119) = −0.59, *p* = 0.56). The interaction between advertising types and green involvement was significant (*b* = 0.42, *t* (119) =2.99, *p* < 0.01). We further probed this interaction by estimating the conditional effects of advertising types on the PIGP at one standard deviation below and above the mean of green involvement. As we expected, participants with lower green involvement (*M* − 1 *SD*) reported a higher PIGP when the advertisement was comparative advertising (4.88) than non-comparative advertisement (4.22), *b* = −0.66, *t* (119) = −3.57, *p* < 0.001. Those with higher green involvement (*M* + 1 *SD*) reported PIGP that did not significantly vary as a function of whether the advertisement was comparative advertising (5.43) or non-comparative advertising (5.55), *b* = 0.12, *t* (119) =0.66, *p* = 0.51 (Figure 6).

### Discussion

Study 4 uses different experimental materials to reveal the moderating effect of green involvement on the comparative advertising effect. Specifically, for consumers with low green involvement, comparative advertising can trigger more positive PIGP than non-comparative advertising. However, for consumers with high green involvement, there is no significant difference in PIGP caused by comparative advertising and non-comparative advertising. From this point of view, comparative advertising plays a more positive role in low-green-involvement consumer groups that lack an understanding of green products and are not familiar with the attributes of green products.

## General discussion

Results of four studies show that comparative advertising affects consumers’ purchase intention of green products by improving their perceived diagnosticity of information. We also identify conditions under which the positive impact of comparative advertising on purchase intention is weakened, namely when the advertising adopts altruistic appeals or the green involvement of the advertising audience is high.

Specifically, the results of Study 1 and 2 indicate that comparative advertising leads to more positive purchase intention of green products than non-comparative advertising, providing evidence for the effectiveness of comparative advertising in the field of green consumption. In Study 2, the mediating role of perceived diagnosticity is also tested. Compared with non-comparative advertising, comparative advertising can effectively enhance consumers’ perceived diagnosticity of information, and thus improve the purchase intention. Study 3 finds that advertising appeals had a moderating effect on the purchase intention of consumers for green products. When advertising expresses egoistic appeal rather than altruistic appeal, using comparative advertising can produce higher perceived diagnosticity and purchase intention than non-comparative advertising. In Study 4, the moderating role of consumers’ green involvement is revealed, and comparative advertising has a more positive effect on those consumers who have low green involvement.

### Theoretical implications

First, our research extends earlier work of comparative advertising. Through reviewing the literature on comparative advertising, we find that the comparative advertising of green products in the current marketing practice has not received enough attention from scholars. Our research introduces comparative advertising into the field of green marketing, and discusses the effectiveness of comparative advertising in the context of green consumption. In addition, based on the Accessibility-diagnosticity theory, we elaborate on the psychological mechanism of comparative advertising to improve consumers’ purchase intention of green products by improving the perceived diagnosticity of information, and explore the boundary conditions of the effect of comparative advertising from the perspective of advertising design dimension (i.e., advertising appeals) and consumer cognition (i.e., green involvement), Our findings further enriched the research on green consumption and comparative advertising.

Second, our research advances earlier studies that provide limited insights into advertising appeals for green products. Regarding the advertising appeals of green products, previous researches have mainly discussed the impact of rational appeal and emotional appeal (Petty et al., 1981; Leonidou et al., 2011), concrete appeal and abstract appeal (Ford et al., 1990; Yang et al., 2015) on consumers’ willingness to buy green products. However, according to the differences between green products and ordinary products, we believe that dividing the advertising appeals of green products into egoistic and altruistic appeal is more helpful to analyze consumers’ psychological cognition and purchase intention of green products. Our research demonstrates that comparative advertising has a more positive effect on consumers’ willingness to buy green products when expressing egoistic appeal but not altruistic appeal.

Third, our findings have implications for research examining the effects of green involvement. Green involvement is a concept extended with the development of green marketing. In recent years, some scholars have discussed the role of consumers’ green involvement in green consumption in different research scenarios (Matthes et al., 2014; Wang et al., 2017). This article discusses the impact of advertising types (comparative vs. non-comparative) on consumers’ purchase intention of green products under different levels of green involvement, and finds that the positive influence of comparative advertising is more obvious among consumers with low green involvement, because they lack experience of green products and are more susceptible to external cues (such as advertising information) when making consumption decisions. On the contrary, consumers with high green involvement have deep processing of green advertisement, and have rich knowledge of green products. Whether it is comparative or non-comparative advertising, they can still rely on their own knowledge and experience to judge green product information.

### Managerial implications

Our findings provide a theoretical reference for the advertising strategy of green product firms. We show that comparative advertising has a positive impact on the willingness to buy green products. Therefore, firms can consider using advertising of indirect comparison according to the attributes of green products, and make comparison with the conventional products familiar to consumers, so as to better convey the information of green products and enhance consumers’ intention to buy. In particular, some newly developed green products may be able to quickly improve product popularity through comparative advertising.

In addition, our findings highlight the key dimension – advertising appeals – when designing comparative advertising for green products. When egoistic appeal is used, comparative advertising leads to significantly higher willingness to buy green products than non-comparative advertising. Therefore, it is not enough for firms to compare altruistic attributes information such as “carbon-emissions-reducing, degradable and environment-friendly” when designing comparative advertising content. In order to give full play to the positive effect of comparative advertising, it is still necessary to highlight the egoistic functional attributes of green products that are different from conventional products, so that consumers can feel the benefits brought by using green products, and firms can really benefit from comparative advertising strategies.

Third, firms should also realize that when consumers have a high degree of green involvement, even well-designed comparative advertising may not be able to effectively improve consumers’ purchase intention of green products. This article shows that consumers’ green involvement influences the effect of comparative advertising, that is, comparative advertising has a stronger effect on those consumers who lack knowledge of green products. Therefore, firms should have clear insight into their target consumers and understand the green involvement of them. For example, firms should actively conduct social listening through social media and other channels to observe consumers’ concerns and comments on public events related to the environment, corporate social responsibility activities and green product advertising. On the other hand, with the popularity of online shopping and the application of big data technology, firms can understand customers’ green involvement by analyzing consumers’ behavior in the stage of collecting green product information, such as the pre-sales dialogue between consumers and online customer service officer.

### Limitations and future research

Based on a variety of brands (real and fictional), manipulation methods and across different product categories, our research provides conclusive evidence for our research hypothesis. However, this article also has some limitations.

First, this research provides consistent evidence to support our hypothesis using different green products as experimental materials (detergent, air conditioner and tissue), which are all utilitarian products. In the future, we can further test whether our findings are still valid for hedonic green products. Researchers can also explore the impact of comparative advertising for different types of green products on consumers based on other classification standards, such as durables and consumables.

Second, we only discuss a limited number of mediators. In addition to perceived diagnosticity, there may be other theories or variables that can explain the effect of comparative advertising on purchase intention of green products. For example, comparative advertising highlights the differences between green products and other products through comparison, which may increase consumers’ willingness to buy by satisfying the need for differentiation or uniqueness.

Finally, our research takes purchase intention as a predictor of green product purchase behavior, and do not directly test the purchase behavior. However, many researchers point out that there is an obvious consumer attitude-behavior gap in green consumption, and consumers generally hold a positive attitude towards green products but seldom take practical actions (White et al., 2019). Further research can investigate and compare how advertising affects different stages of the purchase funnel, especially the actual green consumption behavior of consumers.

## Data availability statement

The raw data supporting the conclusions of this article will be made available by the authors, without undue reservation.

## Ethics statement

The studies involving human participants were reviewed and approved by the Ethics Committee of the School of Management, Jinan University, China. The patients/participants provided their written informed consent to participate in this study. Written informed consent was obtained from the individual(s) for the publication of any potentially identifiable images or data included in this article.

## Author contributions

KN and YLin contributed equally to this work. The tasks they are responsible for consisted of data aggregation, data analysis and interpretation, literature research and writing the initial draft of the manuscript. SY conceived and designed experiments. YLiu and ZL helped in the development of the manuscript in the submitted form and language polishing of the manuscript. All authors contributed to the article and approved the submitted version.

## Funding

The authors acknowledge the financial support provided by the National Social Science Fund of China (No. 22BGL123).

## Conflict of interest

The authors declare that the research was conducted in the absence of any commercial or financial relationships that could be construed as a potential conflict of interest.

## Publisher’s note

All claims expressed in this article are solely those of the authors and do not necessarily represent those of their affiliated organizations, or those of the publisher, the editors and the reviewers. Any product that may be evaluated in this article, or claim that may be made by its manufacturer, is not guaranteed or endorsed by the publisher.

## Footnotes

^1^Credamo is a leading data survey platform in China that provides services such as questionnaire design, distribution and analysis, and helps universities and enterprises with their surveys or researches. Compared with other platforms, Credamo provides multiple screening functions for the recruitment of participants in the experiment, such as refusing participants with low personal credit scores, prohibiting the same IP address from filling in questionnaires repeatedly, intelligent human-computer verification, etc., which helps to reduce invalid experimental data and improve the preciseness of the research.

^2^ https://www.zhizhizhi.com/n/4xahb
